# Deep Brain Stimulation Selection Criteria for Parkinson’s Disease: Time to Go beyond CAPSIT-PD

**DOI:** 10.3390/jcm9123931

**Published:** 2020-12-04

**Authors:** Carlo Alberto Artusi, Leonardo Lopiano, Francesca Morgante

**Affiliations:** 1 Department of Neuroscience ‘Rita Levi Montalcini’, University of Torino, 10126 Torino, Italy; Leonardo.lopiano@unito.it; 2 Neurosciences Research Centre, Molecular and Clinical Sciences Research Institute, St George’s University of London, London SW17 0RE, UK; fmorgante@gmail.com; 3 Department of Clinical and Experimental Medicine, University of Messina, 98125 Messina, Italy

**Keywords:** Parkinson’s disease, deep brain stimulation, selection, levodopa, axial symptoms, non motor symptoms, genetics

## Abstract

Despite being introduced in clinical practice more than 20 years ago, selection criteria for deep brain stimulation (DBS) in Parkinson’s disease (PD) rely on a document published in 1999 called ‘Core Assessment Program for Surgical Interventional Therapies in Parkinson’s Disease’. These criteria are useful in supporting the selection of candidates. However, they are both restrictive and out-of-date, because the knowledge on PD progression and phenotyping has massively evolved. Advances in understanding the heterogeneity of PD presentation, courses, phenotypes, and genotypes, render a better identification of good DBS outcome predictors a research priority. Additionally, DBS invasiveness, cost, and the possibility of serious adverse events make it mandatory to predict as accurately as possible the clinical outcome when informing the patients about their suitability for surgery. In this viewpoint, we analyzed the pre-surgical assessment according to the following topics: early versus delayed DBS; the evolution of the levodopa challenge test; and the relevance of axial symptoms; patient-centered outcome measures; non-motor symptoms; and genetics. Based on the literature, we encourage rethinking of the selection process for DBS in PD, which should move toward a broad clinical and instrumental assessment of non-motor symptoms, quantitative measurement of gait, posture, and balance, and in-depth genotypic and phenotypic characterization.

## 1. Introduction

Despite having being introduced in clinical practice more than 20 years ago, selection criteria for deep brain stimulation (DBS) as an effective treatment for advanced Parkinson’s disease (PD) still rely on the ‘Core Assessment Program for Surgical Interventional Therapies in Parkinson’s Disease’ (CAPSIT-PD) published in 1999 [1]. These criteria were primarily designed to facilitate clinical research, harmonizing the cohorts of clinical trials. However, most of the indications provided in the CAPSIT-PD document were introduced as guidance into the clinical practice of DBS centers worldwide, being extremely useful in supporting the selection of candidates [2]. Twenty years later, these indications could be considered both restrictive and out-of-date, because the knowledge on PD progression, phenotyping, and genotyping has strongly evolved over the last 20 years. Indeed, according to CAPSIT-PD, only 1.6% of PD subjects would be eligible for DBS, rising to 4.5% when applying more flexible criteria [3]. 

Moreover, a growing number of studies have reported novel data on the outcome of DBS in the short- and long-term follow-up and proposed predictors of DBS response [4,5]. However, evidence on how to improve and refine the selection process based on these insights for candidates to DBS is still lacking. Finally, despite the consolidated efficacy of DBS in improving PD cardinal symptoms and motor complications, the factors predicting a successful outcome on activities of daily living (ADL) and quality of life (QoL) have been addressed only by a few studies so far [6].

Advances in understanding the heterogeneity of PD presentation, courses, phenotypes, and genotypes impose a better identification of DBS candidates as a research priority. Additionally, DBS invasiveness, cost, and the possibility of serious adverse events make it mandatory to predict as accurately as possible the clinical outcome when informing the patients about their suitability for surgery. 

Here, we appraised the DBS pre-surgical assessment for PD starting from the original CAPSIT-PD document and addressed the following topics which may impact on the selection process: early versus delayed DBS; the evolution of the levodopa challenge test; the relevance of axial symptoms; new focus on patient-centered outcome measures; the relevance of non-motor symptoms; and a new role for genetics. Our main aim was to highlight current pitfalls and potentialities in the DBS selection process, stimulating future randomized control trials (RCT) to address specific needs. 

## 2. Early Versus Delayed DBS: How Early?

### 2.1. The Standard Rule

The CAPSIT-PD document recommended that a patient considered for interventional surgery should have a diagnosis of idiopathic PD and a minimum disease duration of five years [1]. These requirements were developed to exclude people with atypical parkinsonism, given the absence of benefits and the risk to harm patients with no idiopathic PD [7].

### 2.2. Pros and Cons

The concept of a five-year disease duration has been challenged upon the results of a large RCT published in 2013 (the EARLY-STIM trial) [8]. In this trial demonstrating the superiority of subthalamic (STN) DBS compared to medical therapy alone, patients were included when having a PD diagnosis of ≥4 years, and fluctuations or dyskinesia present for four years or less [8]. 

The EARLY-STIM trial endorsed a conceptual change about the use of DBS for PD favoring a paradigm shift from DBS as the last therapeutic option for advanced disease stages toward an earlier approach for patients experiencing motor complications. This paradigm change is based on three relevant points: (1) the confirmation of DBS safety over the years, even in the long term; (2) the great efficacy of DBS in improving the QoL of patients, even superior to levodopa alone [9]; (3) an earlier intervention could preserve functional capacity. The evidence of efficacy provided by the EARLY-STIM trial led the U.S. Food and Drug Administration (FDA) to extend the DBS indication to patients with a four year PD diagnosis in the presence of at least four months of uncontrolled motor complications [10]. 

The EARLY-STIM trial has triggered discussion as to whether its findings should be translated into clinical practice [11]. Firstly, the shorter disease duration at the time of surgery might pose the risks of including subjects with atypical parkinsonism for which the five-year rule has been developed for CAPSIT-PD. However, in the EARLY-STIM cohort, the mean disease duration of the surgically-treated group was 7.3 ± 3.1 years and only three cases (0.8% of the cohort) were re-diagnosed as non-idiopathic PD eight years after the first randomization and approximately 15 years after diagnosis [12]. However, it should be noted that the issue of a shorter disease duration at the time of surgery might mirror a greater and faster burden of disability which is also associated with specific genotypes associated to PD, such as severe and complex glucocerobrosidase (*GBA*) gene variants [13], which have been associated to poor DBS functional outcome [14].

A related matter is the difficulty in predicting the trajectory of disease progression at such an early stage, either for more benign or rapidly progressing phenotypes [15], with the consequent risk of referring to surgery patients whose motor fluctuations could remain mild for a long time, or vice-versa, those who may develop symptoms non-responsive to DBS and severely impacting ADL and QoL. However, when looking carefully into the EARLY-STIM cohort, all patients had experienced either motor or psychiatric disability due to PD. Accordingly, further analyses on this population have demonstrated that STN-DBS was successful in improving freezing of gait (FoG) in OFF medication condition [16], which affected 52% of the patients at baseline. Remarkably, behavioral complications linked to dopaminergic overmedication had a better outcome in the neurostimulation group [17].

How early should DBS be considered in PD? A pilot open label study on 28 patients suggested to consider DBS even earlier, before motor complications arose [18]. However, despite long term follow-up data on the same cohort [19], the impact of early surgical intervention in such earlier stages is still unknown and should be carefully interpreted including the risks we mentioned above but also a presumptive neuroprotective effect [20]. Finally, it should be taken into account that DBS (STN-DBS in particular) allows a reduction in dose of dopaminergic therapies in most patients [21]. Although favoring the improvement of dyskinesia, the reduction in antiparkinsonian drugs could have role in improving impulsive-compulsive behaviors and obsessive-compulsive and paranoid traits [22,23].

### 2.3. Recommendations

There is evidence for an earlier use of DBS as a treatment option to improve patients’ QoL and early levodopa-responsive axial symptoms, while minimizing the psychiatric consequences of overtreatment. Long-term results from the EARLY-STIM trial would allow the better defining of which PD features are associated to a long-term successful outcome. However, there is not enough knowledge on how early into the disease history DBS should be considered, given the paucity of published data and the current lack of knowledge on how to predict disease progression and DBS response in such early stages. We recommend considering each case singularly, according to the patient’s phenotype, age, needs, and expectations in patients whose symptoms significantly impact ADL and QoL despite a reasonable number of attempts to provide the best medical therapy.

## 3. The Evolution of the Levodopa Challenge Test

### 3.1. The Standard Rule

The second recommendation of CAPSIT-PD is dopaminergic responsiveness confirmed by a levodopa/apomorphine challenge test (LCT). Accordingly, the test has to demonstrate at least a 33% decrease in the Unified Parkinson’s Disease Rating Scale (UPDRS) part III score in the “defined-on condition” (best therapeutic effect after medication agreed by patient and physician) compared to the “defined-off condition” (at least 12 h after receiving the last medication dose). 

### 3.2. Pros and Cons

The threshold of 33% for UPDRS improvement is considered relevant to rule out possible misdiagnoses (i.e., identifying atypical parkinsonism for which DBS is not recommended). The LCT is also important to inform the possible outcome of surgery, showing the likely extent of symptom improvement after surgery, and to establish realistic expectations from DBS [24]. Indeed, it is generally accepted that symptoms improving with levodopa are likely to respond to DBS [25]. However, there are some exceptions and caveats to these widely accepted concepts. Firstly, levodopa-resistant tremor represents one of the indications of DBS, even in the absence of disabling motor fluctuations [26,27], given its excellent effect in controlling or even suppressing tremors, regardless of the deep nuclei targeted [28]. 

Another relevant challenge related to the LCT is the cut-off of 33%. This value has been validated by a study based on its ability to predict chronic levodopa responsiveness, with a positive predictive value for the PD diagnosis of 88.6% [29]. Notably, the Movement Disorders Society (MDS)-sponsored UPDRS scale introduced in 2008 has some differences in the scoring of the part-III (motor part), and a study analyzing the MDS-UPDRS scores with the old UPDRS [30] ones after an acute LCT found an excellent correlation between the two scales, with the 30% UPDRS score variation used for predicting sustained long-term levodopa response equivalent to 24% in MDS-UPDRS [31]. However, data from STN-DBS clinical trials seems to indicate that an excellent response to levodopa (i.e., >50% UPDRS part-III improvement) could be associated with a better DBS motor outcome [32]. A meta-analysis published in 2006 on the STN-DBS outcomes supports this hypothesis, demonstrating that the magnitude of decrease in both UPDRS part-II and part-III scores exhibits a dose–response relationship with the presurgical response to the levodopa challenge test [33]. However, it is unknown whether the magnitude of response at the LCT may predict better ADL and Qol after DBS. Moreover, the relevance of axial symptoms as a source of disability, their heterogenous response to dopaminergic therapies, and their influence on the trajectory of the PD course put in light further considerations on the usefulness of the presurgical LCT simplistically considered as a >30% motor response. 

### 3.3. Recommendations

We recommend using LCT as a key tool to obtain relevant presurgical information on the patient’s status and the possibility of improvement after DBS. However, the rule of UPDRS part-III improvement >30% should not be strictly applied. Although patients affected by disabling dopa-resistant tremor could represent an exception to this rule, improvement >50% can be associated with greater overall benefit in most patients. The LCT response of disabling axial symptoms, such as FoG, is important and should be weighted independently from the percentage UPDRS part-III total score improvement.

## 4. The Relevance of Axial Symptoms: How Sensitive Is Current Clinical Assessment?

### 4.1. The Standard Rule

The term ‘axial symptoms’ is commonly referred to as a group of PD motor features encompassing gait impairment, postural instability, postural abnormalities, and speech disorders, especially dysarthria and stuttering. These are a major source of disability because they are associated with reduced mobility, communication difficulties, recurrent falls, and subsequent injuries [34]. Moreover, they are markers of advanced disease and are often resistant to dopaminergic therapies or exhibit an heterogenous pattern of response to levodopa [35]. There are no precise indications on how to consider these symptoms and their pre-surgical response to levodopa in the clinical practice. 

### 4.2. Pros and Cons

The evidence on the effect of DBS on axial symptoms is controversial. A meta-analysis published in 2004 showed that one year after surgery, STN-DBS or globus pallidus pars interna (GPi) DBS can improve gait and balance symptoms, with an effect size similar to the preoperative effects of dopaminergic medication [36]. However, the improvement provided by DBS seems not sustained over the years. Evidence for axial symptom progression, despite a good control of PD appendicular motor symptoms, has been shown in open-label, long-term follow-up studies, although some extent of axial improvement related to stimulation is reported in the first years after surgery [37,38,39,40]. 

The relevance of axial symptoms as a marker of disease progression was disclosed in a cohort of 143 PD patients treated with STN-DBS [5], in whom axial disability during the follow-up period was strongly associated with an increased risk of death (hazard ratio of 4.3), proving to be the most accurate mortality predictor, even superior to the cognitive status. 

Axial symptoms track disease progression and disability, therefore an accurate presurgical evaluation of levodopa-responsiveness would be necessary for estimating the extent of response after DBS. Indeed, worsening or amelioration after DBS of speech, posture and gait disorders is multifactorial and depends upon clinical variables such as disease duration [16,41], the type of axial symptom (gait often improves after DBS, speech may worsen as a stimulus-related side effect), their interplay with dopaminergic medications [42,43], the brain target employed for DBS, the frequency [44,45] and distribution of stimulation [46], and placement of active electrode contact [41].

Levodopa-resistant axial symptoms are considered a relative contraindication for surgery [47]; however, the current pre-surgical clinical examination is unable to detect early axial signs which may foster worse DBS outcomes. In particular, FoG evaluation poses great challenges, given the episodic nature of this phenomenon and the complex relationship with dopaminergic medications. Trunk postural abnormalities also represent a source of difficulty at the time of DBS selection (including on which target to choose), because camptocormia and Pisa syndrome might be responsive to STN-DBS, even with poor or no amelioration after LCT [48,49].

These difficulties which impact on the selection process might be overcome by integrating objective evaluation involving kinematic analysis and wearable sensors to the pre-operative clinical examination. Specifically, novel technological developments on wearable sensors for home-monitoring have the potential to provide a measure of axial symptoms and their relation to levodopa intake in a naturalistic way [50,51]. These technologies should be employed to detect early axial signs which might predict worsening after DBS and assist the identification of the best candidates. Indeed, a study employing kinematic assessment of gait demonstrated a correlation between presurgical levodopa response of stride length and range of motion and FoG outcome after DBS [52]. 

### 4.3. Recommendations

The global burden of axial symptoms can be considered a proxy for disease stage because of their correlation with disability and death. A fine-grained assessment of each axial symptom, the accurate evaluation of their relationship with dopaminergic therapy, and the integration of technology outcome measures into the clinical practice should favor a better understanding of candidates to DBS, with the potential to predict their disease course and the probability to improve after DBS. Pending clinical trials aiming at the evaluation of the effect of DBS on PD-related axial symptoms, we recommend to accurately evaluate in clinical practice the presence, severity, and impact on patient’s daily life and independence of axial symptoms before surgery, and discuss the weight of each symptom with the patient, clarifying its poor, good, or indeterminate probability of improvement after DBS. Severe FoG and speech issues, in particular, represent a potential challenge in the management of patients undergoing DBS, while camptocormia and Pisa syndrome could have good chances of improvement and should not be considered contraindications for DBS. 

## 5. The Need for Patient-Centered Outcome Measures

### 5.1. The Standard Rule

From a regulatory point of view, the FDA and European Medicine Agency (EMA) request the presence of motor fluctuations as a mandatory criterion for DBS indication in PD [10]. Reduction in severity and frequency of motor fluctuations represents one of the most relevant achievements obtained by DBS, which translates into the improvement of QoL revealed by randomized controlled trials [8,53]. 

### 5.2. Pros and Cons

In CAPSIT-PD, it is recommended that the patients perform the self-reporting diary one week per month during the three preoperative months, indicating the presence of four conditions: complete OFF, partial OFF, complete ON, and ON with dyskinesias [1]. However, these measures are highly subjective, and wrong or missed entries may occur in about one third of cases—also when using electronic motor diaries [54]. That is, objective home-based quantification by wearable sensors of PD motor symptoms [55], including FoG [56], should be explored carefully in patients considered for DBS, also with respect to the predictive value of these measures. Indeed, when it comes to predicting the outcome of DBS in PD and patient-centered outcome measures are employed, some discrepancies arise. Patient-centered outcome measures are represented by QoL, evaluated by the validated Parkinson’s Disease Questionnaire 39 (PDQ-39) [57] or by its short form (PDQ-8) [58], and ADL functioning or independence, typically measured by UPDRS part-II and the Schwab and England (S&E) scale [59]. The importance of measuring patient-centered outcomes relates to the discrepancy between the judgment made in-clinic by the neurologist and the degree of satisfaction [60] and independence obtained by the patient during daily life [54]. Two key factors may account for this discrepancy: (1) motor symptoms observed by clinicians explain only a small part of the complex picture of PD, which encompasses several non-motor symptoms; (2) the standardized tasks assessed during in-clinic visits and the non-quantitative, non-continuous, non-ecologic in-clinic examinations may not represent a comprehensive measure of the patient situation and condition during daily life. This last aspect is true even when limiting the evaluation to motor symptoms, in particular episodic motor symptoms such as FoG [61], which are not adequately captured during in-clinic standard assessments [54]. 

When it comes to analyzing determinants of improvement in QoL after STN DBS, a post-hoc analysis of the EARLY-STIM trial found smaller QoL improvement at 24-months follow-up in patients with better pre-surgical PDQ-39 scores [6]. Interestingly, patients with pre-surgical PDQ-39 scores ≤ 15 had no significant change in QoL following surgery. This finding is not meant to be caused by a ceiling effect of STN-DBS to improve motor symptoms in the EARLY-STIM cohort, because the change in QoL over the two years was independent of the severity of parkinsonian motor signs assessed by UPDRS-III [8]. Accordingly, in another study analyzing a cohort of 85 PD patients treated with DBS, the magnitude of motor symptom improvement with a pre-surgical LCT was only borderline associated with improvement of QoL after DBS (*p* = 0.053) [62].

A systematic review [63] demonstrated that higher baseline QoL predicted larger QoL changes after surgery in three out of four studies. The analysis of the 18 studies included in this review yielded mixed results with respect to the predictive value of other clinical and demographical features. There are two main explanations for such discrepant findings: (1) most of these studies were not primarily designed to detect predictors of QoL change after DBS and results are influenced by the main a priori hypothesis tested in each study; (2) factors contributing to QoL in PD include not only motor disability but also non-motor symptoms [64], and the interplay between these domains might be individualized and have a different weight in each subject. Moreover, among motor symptoms, axial disability (and its response to therapies) might have a high impact, which has not been yet explored carefully in regard to QoL or ADL outcome after DBS. 

### 5.3. Recommendations

Future studies should be designed to capture predictors of QoL and ADL improvements after DBS, taking into account the heterogeneity of the disease, the contribution of non-motor symptoms, and the impact of axial symptoms. We recommend evaluating both the ADL and QoL of candidates for DBS by means of validated scales (e.g., UPDRS part-II and PDQ-39 or PDQ-8) and carefully discuss with patients the disease burden and their determinants. After surgery, these scales can inform more than the in-clinic motor assessment about the clinical status of the patient and the impact of stimulation on their functioning in daily life, guiding possible changes in stimulation parameters, medical therapy or non-medical interventions, such as psychological support, physiotherapy, and emotional and social stimulation. 

## 6. The Complexity of PD Spectrum Integrated into the Selection Process: Relevance of Non-Motor Symptoms

### 6.1. The Standard Rule

Despite the fact that DBS was developed to treat motor symptoms, the growing relevance given to non-motor symptoms (NMS) in the last 15 years fostered investigations into the effect of DBS for these features as well [65,66]. There are no indications nor clues so far on how to consider the presence and burden of non-motor symptoms in PD candidates for DBS. 

### 6.2. Pros and Cons

A few studies have demonstrated the improvement of different NMS (cardiovascular, sleep/fatigue, perceptual problems/hallucinations, gastrointestinal, urinary, and miscellaneous domains) six months after surgery [65], which were maintained at 24 months for the sleep/fatigue, urinary and miscellaneous domains [66], and at 36 months for the sleep domain [67]. These findings were confirmed in a small cohort of young onset PD patients, for whom STN-DBS provided sustained improvement of the sleep domain of the Non-Motor Symptoms Scale and Parkinson’s disease sleep scale-2 up to 24 months and correlated to the decrease in dopamine-agonist medication [68]. 

Remarkably, change in NMS frequency and severity after STN-DBS is strongly correlated to the improvement in QoL both in uncontrolled [66] and controlled studies [67,69] performed in the same STN-treated cohort at different follow-up.

In the attempt to define profiles for the best DBS candidates which may encompass the complexity of PD clinical spectrum and its heterogeneity, a new data-driven approach to PD, supported by biomarkers and neuropathology, disclosed three different PD subtypes: mild-motor predominant, intermediate, and diffuse malignant [70,71]. These three groups, defined based on the progression of disability and mortality, differ in the presentation of motor and non-motor symptoms at onset, in particular for the contribution of three types of NMS: cognitive impairment, rapid eye movement sleep behavior disorder, and dysautonomia. When the same subtyping criteria were applied to a cohort of STN-DBS patients at the time of the surgical selection, the mild phenotype seem to perform better on ADL independence at the short and long-term follow-up compared to the malignant phenotype, despite similar efficacy of stimulation on motor symptoms, fluctuations, and ambulatory capacity [72]. 

### 6.3. Recommendations

More efforts are needed to understand which NMS are predictors of good or poor outcome, how different targets of DBS (STN or GPi) should be indicated to treat different NMS based on the ability to decrease total LEDD and dopamine-agonists LEDD [17], or directly treat particular symptoms by means of the stimulation of specific networks involved in pain or mood, apathy and attention [73]. We recommend to carefully assess the presence and severity of non-motor symptoms before surgery and explain to the patients that when the disease burden is mainly driven by non-motor symptoms, DBS might not be the best therapeutic option to consider. 

## 7. A New Role for Genetics

### 7.1. The Standard Rule

One of the most remarkable advances in our understanding of PD pathogenesis in the last 20 years is represented by genetics. The increasing power of genetic analyses led to the identification of several chromosomal loci that cause or modulate the risk for PD [74]. Moreover, specific genetic mutations have been associated to specific clinical features and different disease courses, which could have an impact on the selection for DBS. 

That is, only some evidence on the differential DBS response in different forms of monogenic PD has been put forward, suggesting some differences in the magnitude of response [14,75,76,77]. However, in this case there are no specific recommendations on the use of genetics in the clinical practice of DBS centers. 

### 7.2. Pros and Cons

The main advantage of knowing the genotype of a PD patient appraised for DBS is related to the knowledge of disease evolution associated to a particular gene variant. However, despite the effort of systematic reviews [76,77] and one meta-analysis [14], the small size of the cohorts reported and the paucity of data on different gene variants and brain targets other than STN, do not allow researchers to reach firm conclusions. For example, *LRRK2*-G2019S variant carriers, described in 44 out of 50 *LRRK2* subjects with DBS reported in the literature, show an excellent response to STN DBS, which is also the most reported target [76]. G2019S is the most frequent *LRRK2* variant and produces a phenotype overlapping to late-onset, non-mutated PD with frequent presence of tremor and good response to dopaminergic medications [78]. However, in three out of four reported cases with *LRRK2*-R1441G variant, a mutation variant rarely found outside northern Spain, poor response to STN-DBS was reported [76]. The paucity of data characterizing the phenotype of *LRRK2*-R1441G variant makes it impossible to assume that the poor DBS outcome was due to more severe disease progression and development of DBS resistant features. A similar issue applies to carriers of glucocerebrosidase (*GBA*) gene variants which have a high prevalence of neuropsychiatric symptoms, especially impulsive compulsive behavior and hallucinations, and a higher risk to develop early over disease course cognitive disturbances [13]. Indeed, *GBA*-associated PD showed worse cognitive and functional performances and lower reductions in dopaminergic medication after surgery [14]. However, it is unknown which variants mostly contribute to this result. Remarkably, the risk of hallucinations and cognitive impairment, as well as survival, differs across *GBA* subjects, being higher in subjects carrying complex and severe variants [13,79].

### 7.3. Recommendations

**Figure 1:**
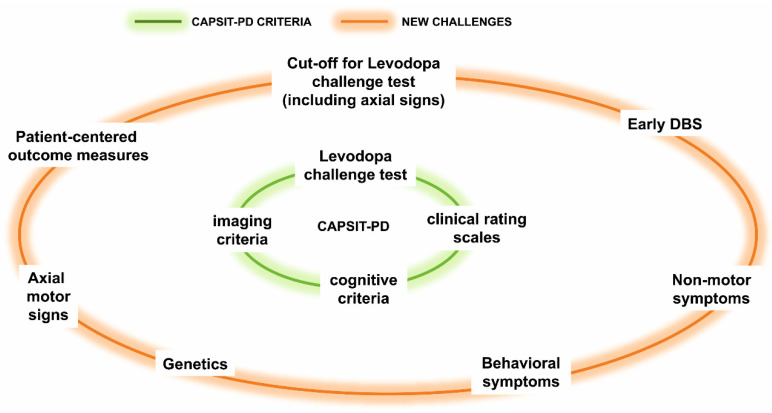
Core Assessment Program for Surgical Interventional Therapies in Parkinson’s Disease (CAPSIT-PD) and recommendations on challenging areas related to the deep brain stimulation (DBS) selection.

Genetic testing is becoming accessible and affordable in clinical practice in many countries and may be used to inform PD candidates for their suitability for DBS. Evidence is still weak to opt for either endorsing DBS or not for a certain patient only relying on the genetic background, but in the future it is probable that certain genotypes will be considered not suitable for DBS on the basis of their improbability to benefit from the effects. To date, genetic testing of patients undergoing DBS might be proposed in those manifesting specific phenotype features (e.g., rapid development of disability, susceptibility to behavioral complications and hallucinations) consistent with particular gene variants, such as severe *GBA* mutations, which may determine possible issues presented in the post-operative follow-up (Figure 1). 

## 8. Conclusions

Advances in understanding both the complexity of PD and the effect of DBS in PD patients have provided new evidence for a better stratification of patients and a more conscious use of this therapeutic option. 

It is critical to take into account that the multifaceted symptomatology of PD, encompassing motor, non-motor, and behavioral issues, makes a candidate’s selection for advanced therapies a process difficult to fit in fixed and precise borders. Additionally, the probability of improving or worsening certain symptoms according to clinical trials (or to post-hoc analysis of trials) cannot capture the full clinical complexity and heterogeneity of the PD clinical spectrum and should be used cautiously for making a decision at a single level. To date, in addition to studies supporting a better comprehension of pre-surgical predictors of DBS outcomes, we also need a shift in the statistical approach to improve the decision-making from a group to an individual level. 

In conclusion, the improvement in the stratification of PD patients according to their clinical features and genetic background can inform the disease course, and more-in-depth knowledge on patients most probable to benefit from DBS. A redefinition of CAPSIT-PD criteria for DBS should be pursued based on the new knowledge gained on PD clinical spectrum and DBS long term follow-up studies. This would allow surgical centers to be more accurate in predicting the outcome after functional neurosurgery and choosing the best target for stimulation. We can now estimate the probability to improve specific disabling symptoms and choose integrated approaches, combining stimulation of specific targets according to patients’ issues (e.g., ventral STN for high non-motor burden [73]) with other therapeutic options (e.g., rehabilitation), with the ultimate goal of improving ADL, mental wellbeing, and eventually the QoL of PD patients. This viewpoint provided updated recommendations for a more accurate and fine-grained assessment of PD patients considered as potential candidates for DBS and highlighted the need for further studies to strengthen the evidence on predictors of DBS outcomes at an individual level, encompassing the complex and multifaceted syndromic picture of the disease and the new possibilities offered by validated clinical scales, technological devices, and genetic analysis.
